# Statin-Associated Muscular and Renal Adverse Events: Data Mining of the Public Version of the FDA Adverse Event Reporting System

**DOI:** 10.1371/journal.pone.0028124

**Published:** 2011-12-20

**Authors:** Toshiyuki Sakaeda, Kaori Kadoyama, Yasushi Okuno

**Affiliations:** 1 Center for Development of Integrative Education in Pharmacy and Pharmaceutical Sciences, Graduate School of Pharmaceutical Sciences, Kyoto University, Kyoto, Japan; 2 Department of Systems Biosciences for Drug Discovery, Graduate School of Pharmaceutical Sciences, Kyoto University, Kyoto, Japan; University of British Columbia, Canada

## Abstract

**Objective:**

Adverse event reports (AERs) submitted to the US Food and Drug Administration (FDA) were reviewed to assess the muscular and renal adverse events induced by the administration of 3-hydroxy-3-methylglutaryl coenzyme A (HMG-CoA) reductase inhibitors (statins) and to attempt to determine the rank-order of the association.

**Methods:**

After a revision of arbitrary drug names and the deletion of duplicated submissions, AERs involving pravastatin, simvastatin, atorvastatin, or rosuvastatin were analyzed. Authorized pharmacovigilance tools were used for quantitative detection of signals, i.e., drug-associated adverse events, including the proportional reporting ratio, the reporting odds ratio, the information component given by a Bayesian confidence propagation neural network, and the empirical Bayes geometric mean. Myalgia, rhabdomyolysis and an increase in creatine phosphokinase level were focused on as the muscular adverse events, and acute renal failure, non-acute renal failure, and an increase in blood creatinine level as the renal adverse events.

**Results:**

Based on 1,644,220 AERs from 2004 to 2009, signals were detected for 4 statins with respect to myalgia, rhabdomyolysis, and an increase in creatine phosphokinase level, but these signals were stronger for rosuvastatin than pravastatin and atorvastatin. Signals were also detected for acute renal failure, though in the case of atorvastatin, the association was marginal, and furthermore, a signal was not detected for non-acute renal failure or for an increase in blood creatinine level.

**Conclusions:**

Data mining of the FDA's adverse event reporting system, AERS, is useful for examining statin-associated muscular and renal adverse events. The data strongly suggest the necessity of well-organized clinical studies with respect to statin-associated adverse events.

## Introduction

Cardiovascular disease (CVD) involves a wide range of disorders, such as ischemic heart disease, heart attack and stroke, and a high level of LDL-cholesterol (LDL-C) in blood is a risk factor for CVD [1]–[5]. Given that a reduction in LDL-C results in the prevention of CVD, the inhibitors of 3-hydroxy-3-methylglutaryl coenzyme A (HMG-CoA) reductase (statins) are currently used for the primary and secondary prevention of CVD [1]–[5]. Recently, it has been suggested that a more intensive lowering of LDL-C could achieve better clinical benefits, and rosuvastatin has attracted attention [6], [7]. However in 2003, controversial concerns were raised about its safety in a respected international journal, in terms of rhabdomyolysis and renal failure, on the basis of premarketing studies and post-marketing reports [8]–[14]. The continuous debate about rosuvastatin, and withdrawal of another potent statin, cerivastatin, from the global market have posed a variety of problems concerning pharmacovigilance [15], [16].

In 2005 and 2006, two post-marketing analyses were published [17], [18], in which the safety of statins was assessed using adverse event reports (AERs) submitted to the US Food and Drug Administration (FDA). This database relies on reports of spontaneous adverse events to the FDA generated by health professionals, consumers, and manufacturers, and the system is referred to as the Adverse Event Reporting System (AERS). Despite the small number of AERs at that time, the reports provided information valuable for clinical decisions, because it was user-derived. Continuous operation of the AERS thereafter has resulted in an enormous database, and in this study, about 2 million AERs submitted to the AERS from 2004 to 2009 were reviewed to assess the muscular and renal adverse events induced by the administration of statins and to attempt to determine their rank-order of the association. To evaluate the results statistically, authorized pharmacovigilance methods were used for quantitative signal detection [19]–[25], where a signal means a drug-associated adverse event. Here, the AERs with pravastatin, fluvastatin, lovastatin, simvastatin, atorvastatin, and rosuvastatin were analyzed, and myalgia, rhabdomyolysis and an increase in creatine phosphokinase level were focused on as the muscular adverse events, and acute renal failure, non-acute renal failure, and an increase in blood creatinine level as the renal adverse events.

## Methods

### Data sources

Input data for this study were taken from the public release of the FDA's AERS database, which covers the period from the first quarter of 2004 through the end of 2009. The data structure of AERS is in compliance with international safety reporting guidance, ICH E2B, consisting of 7 data sets; patient demographic and administrative information (DEMO), drug/biologic information (DRUG), adverse events (REAC), patient outcomes (OUTC), report sources (RPSR), drug therapy start and end dates (THER), and indications for use/diagnosis (INDI). The adverse events in REAC are coded using preferred terms (PTs) in the Medical Dictionary for Regulatory Activities (MedDRA) terminology. Here, version 13.0 of MedDRA was used.

Prior to analysis, all drug names were unified into generic names by a text-mining approach, because AERS permits the registering of arbitrary drug names, including trade names and abbreviations. Spelling errors were detected by GNU Aspell and carefully confirmed by working pharmacists. Foods, beverages, treatments (e.g. X-ray radiation), and unspecified names (e.g., beta-blockers) were omitted for this study. Duplicated reports were deleted according to FDA's recommendation of adopting the most recent CASE number (as described in one of the downloaded files, ‘Asc_nts.doc’ from the web-site of the FDA AERS database), resulting in the reduction of the number of AERs from 2,231,029 to 1,644,220. The total number of co-occurrences, i.e., drug-adverse event pairs, was 22,017,956.

### Definition of adverse events

As the muscular adverse events, myalgia, rhabdomyolysis and an increase in creatine phosphokinase level were focused herein, and these events are coded by preferred terms (PTs) as PT10028411, PT10039020, and PT10005470, respectively, by MedDRA version 13.0, in which 18, 2 and 12 lower level of terms (LLTs) are assigned, respectively. For example, muscle pain (LLT10028322) and tenderness muscle (LLT10043230) are included in myalgia (PT10028411). Additionally, statin-associated asthenia (PT10003549, 22 LLTs), chest pain (PT10008479, 38 LLTs), pain in the extremities (PT10033425, 39 LLTs), muscle spasms (PT10028334, 38 LLTs), muscular weakness (PT10028372, 23 LLTs), myositis (PT10028653, 7 LLTs), and myopathy (PT10028641, 12 LLTs) were analyzed. As for the renal adverse events, acute renal failure (PT10038436, 28 LLTs), non-acute renal failure (PT10038435, 12 LLTs), and an increase in blood creatinine level (PT10005483, 9 LLTs) were focused.

### Data mining

In pharmacovigilance analyses, data mining algorithms have been developed to identify drug-associated adverse events as signals that are reported more frequently than expected by estimating expected reporting frequencies on the basis of information on all drugs and all events in the database [23]–[25]. For example, the proportional reporting ratio (PRR) [19], the reporting odds ratio (ROR) [20], the information component (IC) [21], and the empirical Bayes geometric mean (EBGM) [22] are widely used, and indeed, the PRR is currently employed by the Medicines and Healthcare products Regulatory Agency (MHRA), UK, the ROR by the Netherlands Pharmacovigilance Centre, the In by the World Health Organization (WHO), and the EBGM by the FDA.

All of these algorithms extract decision rules for signal detection and/or calculate scores to measure the associations between drugs and adverse events from a two-by-two frequency table of counts that involve the presence or absence of a particular drug and a particular event occurring in case reports. These algorithms, however, differ from one another in that the PRR and ROR are frequentist (non-Bayesian), whereas the IC and EBGM are Bayesian. In this section, only the scoring thresholds used in the present study are given, and the reader is referred to review articles for more extensive details of each statistical test [23]–[25].

Here, we define how a drug and associated adverse event is classified as a signal when using each statistical test. Using the PRR, a signal is detected, if the count of co-occurrences ≥3 and the PRR≥2.0 with an associated χ^2^ value≥4.0 [19]. For the ROR, a signal is detected, if the lower bound of the 95% two-sided confidence interval exceeds 1 [20]. Signal detection using the IC is done using the IC025 metric, a criterion indicating the lower bound of the 95% two-sided confidence interval of the IC, and a signal is detected with the IC025 value exceeds 0 [21]. Finally, the EB05 metric, a lower one-sided 95% confidence limit of EBGM, is used and a signal is detected when EB05 is greater than or equal to the threshold value 2.0 [22]. In this study, the adverse events were extracted when at least 1 of 4 indices met the criteria indicated above.

## Results

The total number of co-occurrences with pravastatin was 53,317, and 16,527 for fluvastatin, 21,345 for lovastatin, 180,042 for simvastatin, 220,194 for atorvastatin, and 57,389 for rosuvastatin, representing 0.242%, 0.075%, 0.097%, 0.818%, 1.000% and 0.260% of all co-occurrences in the database, respectively. In total, 701, 628, 490, 744, 883 and 619 adverse events were extracted as statin-associated adverse events with 17,815, 5,469, 8,345, 82,028, 100,133, and 30,356 co-occurrences, respectively. The total number of adverse events occurring with fluvastatin and lovastatin was not large enough to compare the association with adverse events.

The signals for myalgia, rhabdomyolysis, and an increase in creatine phosphokinase level were detected for pravastatin, simvastatin, atorvastatin, and rosuvastatin. The statistical data are listed in Table 1. Although the signals were detected for these statins, the association with myalgia was noteworthy for rosuvastatin. As for rhabdomyolysis and the increase in creatine phosphokinase level, statistical indices indicated a stronger association for simvastatin and rosuvastatin. Other muscular adverse events found commonly for these four statins included asthenia, chest pain, pain in the extremities, muscle spasms, muscular weakness, myositis, and myopathy, and a stronger association was found for rosuvastatin (statistical data not shown).

**Table 1:** Signal detection for statin-associated muscular adverse events.

	Statins	N	PRR (kai2)	ROR (95% CI)	IC (95% CI)	EBGM (95% CI)
Myalgia	Pravastatin	518	3.047 (704.853)*	3.062 (2.807, 3.316)*	1.591 (1.465, 1.716)*	3.000 (2.789)*
	Simvastatin	1980	3.453 (3437.911)*	3.524 (3.369, 3.678)*	1.774 (1.710, 1.839)*	3.418 (3.293)*
	Atorvastatin	2456	3.503 (4383.844)*	3.593 (3.450, 3.735)*	1.795 (1.737, 1.853)*	3.468 (3.354)*
	Rosuvastatin	1693	9.439 (12420.824)*	9.646 (9.185, 10.107)*	3.193 (3.122, 3.263)*	9.186 (8.825)*
Rhabdomyolysis	Pravastatin	212	2.246 (145.068)*	2.253 (1.968, 2.538)*	1.152 (0.958, 1.347)*	2.205 (1.967)
	Simvastatin	2278	7.210 (12122.472)*	7.594 (7.278, 7.911)*	2.830 (2.769, 2.891)*	7.129 (6.887)*
	Atorvastatin	1114	2.861 (1353.202)*	2.915 (2.746, 3.084)*	1.509 (1.423, 1.595)*	2.840 (2.703)*
	Rosuvastatin	605	5.994 (2492.472)*	6.073 (5.602, 6.544)*	2.558 (2.442, 2.674)*	5.933 (5.546)*
Increase of CPK	Pravastatin	206	2.461 (177.003)*	2.470 (2.153, 2.787)*	1.283 (1.085, 1.480)*	2.410 (2.147)*
	Simvastatin	1036	3.673 (2017.111)*	3.755 (3.529, 3.981)*	1.866 (1.777, 1.956)*	3.641 (3.458)*
	Atorvastatin	997	2.886 (1233.942)*	2.942 (2.762, 3.122)*	1.522 (1.431, 1.613)*	2.865 (2.719)*
	Rosuvastatin	505	5.634 (1908.004)*	5.702 (5.220, 6.184)*	2.469 (2.342, 2.596)*	5.581 (5.182)*

N: the number of co-occurrences.

PRR: the proportional reporting ratio [19], ROR: the reporting odds ratio [20], IC: the information component [21], EBGM: the empirical Bayes geometric mean [22].

CI: the confidence interval; two-sided for ROR and IC, and one-sided for EBGM.

*: signal detected, see “Methods” for the criteria of detection.

Myalgia, rhabdomyolysis and increase of creatine phosphokinase (CPK) level were coded as PT10028411, PT10039020 and PT10005470, respectively.

The data concerning acute renal failure, non-acute renal failure, and an increase in blood creatinine level are listed in Table 2. Acute renal failure was associated with all 4 statins, though in the case of atorvastatin, the association was marginal, and furthermore, a signal was not detected for non-acute renal failure or for an increase in blood creatinine level.

**Table 2:** Signal detection for statin-associated renal adverse events.

	Statins	N	PRR (kai2)	ROR (95% CI)	IC (95% CI)	EBGM (95% CI)
Acute renal failure	Pravastatin	338	1.424 (42.101)	1.426 (1.281, 1.570)*	0.503 (0.349, 0.658)*	1.414 (1.292)
	Simvastatin	1371	1.713 (406.242)	1.723 (1.633, 1.813)*	0.771 (0.693, 0.848)*	1.704 (1.630)
	Atorvastatin	1112	1.133 (17.315)	1.135 (1.069, 1.200)*	0.179 (0.093, 0.264)*	1.131 (1.077)
	Rosuvastatin	340	1.330 (27.468)	1.332 (1.197, 1.466)*	0.406 (0.252, 0.560)*	1.322 (1.209)
Non-acute renal failure	Pravastatin	237	1.160 (5.056)	1.160 (1.021, 1.300)*	0.209 (0.025, 0.393)*	1.153 (1.036)
	Simvastatin	817	1.184 (23.316)	1.186 (1.107, 1.265)*	0.242 (0.142, 0.341)*	1.182 (1.115)
	Atorvastatin	Not detected				
	Rosuvastatin	299	1.361 (28.138)	1.362 (1.215, 1.509)*	0.438 (0.274, 0.602)*	1.351 (1.228)
Increase of CR	Pravastatin	242	1.635 (58.964)	1.638 (1.443, 1.833)*	0.700 (0.518, 0.883)*	1.618 (1.454)
	Simvastatin	629	1.257 (33.035)	1.260 (1.165, 1.355)*	0.328 (0.214, 0.441)*	1.254 (1.174)
	Atorvastatin	Not detected				
	Rosuvastatin	196	1.229 (8.134)	1.230 (1.069, 1.391)*	0.291 (0.089, 0.494)*	1.220 (1.084)

N: the number of co-occurrences.

PRR: the proportional reporting ratio [19], ROR: the reporting odds ratio [20], IC: the information component [21], EBGM: the empirical Bayes geometric mean [22].

CI: the confidence interval; two-sided for ROR and IC, and one-sided for EBGM.

*: signal detected, see “Methods” for the criteria of detection.

Acute renal failure, non-acute renal failure and increase of blood creatinine (CR) level were coded as PT10038436, PT10038435 and PT10005483, respectively.

## Discussion

The PRIMO study, an observational study of muscular symptoms in an unselected population of about 8000 hyperlipidemic patients receiving high doses of statins, indicated that the symptoms were reported by 10.5% of patients [3], [26]. In a recently published review, it was suggested that the muscular symptoms occurred in up to 20% of patients in observational studies [27], and Dirks and Jones indicated that as many as 25% of statin users who exercise may experience muscular symptoms [28]. The prevalence varied among the reports, and this can be explained, in part, by ambiguity in the terms [4]. The National Lipids Association's Muscle Expert Panel and other statin experts have emphasized the importance of standardizing terms in order to allow reliable comparisons among studies and to improve care for statin users [4]. Generally, myopathy or myalgia is the term to describe all muscular symptoms [4], [27]. If it is accompanied by elevation in creatine phosphokinase level, the condition is known as myositis [4], but myositis does not always require such conditions [27]. The severe case is understood to be rhabdomyolysis [4], [27].

Golomb et al. pointed out the importance of physician response to patient reports of statin-associated adverse events [29]. Using a patient-targeted survey, they indicated that 87% of patients reportedly spoke to their physicians about the possible connection between statin use and symptoms, but physicians were more likely to deny than affirm the possibility [29]. The AERS database covers several million case reports on adverse events, and is characterized by spontaneity. Pharmacovigilance aims to search for previously unknown patterns and automatically detect important signals, i.e., drug-associated adverse events, from such a large database. Recently developed data mining tools, i.e., the PRR, ROR, IC, and EBGM, have been successful at detecting signals that could not be found by individual case reviews and that warrant further investigation together with continuous surveillance. These tools are now used routinely for pharmacovigilance, supporting signal detection and decision-making at companies, regulatory agencies, and pharmacovigilance centers [19]–[25]. Comparisons of specificity have showed that none of these indices is universally better than the others [20], [23], [24].

The AERS database is considered a valuable tool; however, some limitations inherent to spontaneous reporting have been pointed out [23]. First, the data occasionally contain misspelling and miswords, although the structure of AERS is in compliance with the international safety reporting guidance. Second, the system was started more than 10 years ago, and reporting patterns have changed over time. Third, the adverse events are coded using hierarchical terms of PTs of MedDRA, and changes in terminology over time also might affect the quality of the database. Last, there are a number of duplicate entries in the database. To overcome problems with data quality, we manually corrected mistakes in the data entities and deleted duplicates according to FDA's recommended method.

Here, it was suggested that the muscular adverse events were associated with pravastatin, simvastatin, atorvastatin, and rosuvastatin, including myalgia, rhabdomyolysis, an increase in creatine phosphokinase level, asthenia, chest pain, pain in extremities, muscle spasms, muscular weakness, myositis and myopathy. Additionally, according to the PRR, ROR, IC and EBGM values, these muscular adverse events were more noteworthy for rosuvastatin than pravastatin and atorvastatin. This was not correlated with the rank-order of inhibitory activity of HMG-CoA reductase, suggesting that these adverse events were, in part, independent of cholesterol metabolic pathways. These data strongly suggest the necessity of randomized controlled clinical studies or observation cohort studies with respect to statin-associated muscular adverse events. Also, acute renal failure was associated with 4 statins, but the association was marginal for atorvastatin. It should be noted that no signals were detected for atorvastatin-associated non-acute renal failure and an increase in blood creatinine level. These results are not contradictory to the findings of the large-scale clinical studies GREACE [30], TNT [31], CARDS [32] and ALLIANCE [33], in which atorvastatin was found to improve renal function.

There is no credible counterfactual, e.g., randomized control group, to extract the drug-associated adverse events as signals, and therefore the disease-oriented adverse events can be extracted as signals. For example, myalgia was extracted as a statin-associated adverse event for pravastatin, simvastatin, atorvastatin, and rosuvastatin, but this adverse event might also be commonly found in hyperlipidemic patients irrespective of administration of statins. It is noted that the results can be biased by unmeasured confounding factors. Although the comparison among statins possibly offsets them, a statistically well-organized methodology should be established to minimize their effects.

Advanced age and female sex are risk factors for statin-associated adverse events [27], [34]–[36]. The DEMO file of AERS data includes patient demographic and administrative information. Age data was valuable for 1,084,999 (66.0%) of 1,644,220 AERs; the average (±SD) was 52.7±23.2 years. The gender was clarified in 1,520,994 AERs (92.5%), and the ratio was male/female/unknown = 605,271/915,723/123,226 (36.8%/55.7%/7.5%). There were no statistically significant differences of age and gender among statins. Again, there was no rational method to elucidate the risk factors for drug-associated adverse events, and additional tools should be established to exploit the data herein.

In conclusion, AERs submitted to the FDA were reviewed to assess the statin-associated muscular and renal adverse events and to attempt to determine the rank-order of the association. Based on 1,644,220 AERs from 2004 to 2009, it was suggested that the adverse events, including myalgia, rhabdomyolysis, an increase in creatine phosphokinase level and other muscular events, were associated with pravastatin, simvastatin, atorvastatin, and rosuvastatin, and these events were more noteworthy for rosuvastatin than pravastatin and atorvastatin. Acute renal failure was also associated with 4 statins, but the association was marginal for atorvastatin. These data strongly suggest the necessity of well-organized clinical studies with respect to statin-associated adverse events.
